# Qmatey: an automated pipeline for fast exact matching-based alignment and strain-level taxonomic binning and profiling of metagenomes

**DOI:** 10.1093/bib/bbad351

**Published:** 2023-10-11

**Authors:** Alison K Adams, Brandon D Kristy, Myranda Gorman, Peter Balint-Kurti, G Craig Yencho, Bode A Olukolu

**Affiliations:** Department of Entomology and Plant Pathology, University of Tennessee, Knoxville, TN 37996, USA; UT-ORNL Graduate School of Genome Science and Technology, University of Tennessee, Knoxville, TN 37996, USA; Department of Integrative Biology, Michigan State University, East Lansing, MI, USA; W.K. Kellogg Biological Station, Michigan State University, Hickory Corners, MI, USA; Department of Animal Science, University of Tennessee, Knoxville, TN 37996, USA; College of Veterinary Medicine, University of Tennessee, Knoxville, TN 37996, USA; Department of Entomology and Plant Pathology, NC State University, Raleigh, NC 27695-7613, USA; Plant Science Research Unit, USDA-ARS, Raleigh, NC, USA; Department of Horticultural Science, NC State University, Raleigh, NC 27695-7609, USA; Department of Entomology and Plant Pathology, University of Tennessee, Knoxville, TN 37996, USA; UT-ORNL Graduate School of Genome Science and Technology, University of Tennessee, Knoxville, TN 37996, USA

**Keywords:** metagenomics, microbiome, reduced representation sequencing, exact matching, MegaBLAST

## Abstract

Metagenomics is a powerful tool for understanding organismal interactions; however, classification, profiling and detection of interactions at the strain level remain challenging. We present an automated pipeline, quantitative metagenomic alignment and taxonomic exact matching (Qmatey), that performs a fast exact matching-based alignment and integration of taxonomic binning and profiling. It interrogates large databases without using metagenome-assembled genomes, curated pan-genes or k-mer spectra that limit resolution. Qmatey minimizes misclassification and maintains strain level resolution by using only diagnostic reads as shown in the analysis of amplicon, quantitative reduced representation and shotgun sequencing datasets. Using Qmatey to analyze shotgun data from a synthetic community with 35% of the 26 strains at low abundance (0.01–0.06%), we revealed a remarkable 85–96% strain recall and 92–100% species recall while maintaining 100% precision. Benchmarking revealed that the highly ranked Kraken2 and KrakenUniq tools identified 2–4 more taxa (92–100% recall) than Qmatey but produced 315–1752 false positive taxa and high penalty on precision (1–8%). The speed, accuracy and precision of the Qmatey pipeline positions it as a valuable tool for broad-spectrum profiling and for uncovering biologically relevant interactions.

## INTRODUCTION

Metagenomics facilitates the high-throughput study of micro-ecosystems based on the analysis of DNA sequence data derived from a community of organisms [1]. While the organisms usually profiled are prokaryotes and fungi, there is growing interest in other groups of unicellular and multicellular organisms, such as viruses, protists, nematodes and insects that play critical roles within communities. Metagenomic sequencing is being used to understand the functional roles microbes play in the health and productivity of hosts and ecosystems. This requires identifying host–microbiome, microbe–microbe and multipartite interactions, which in turn requires accurate strain level profiling [2]. For example, pathogenicity is strain-specific in many species and often modulated by intra-specific and inter-specific interactions [3]. As the development of high-throughput technologies and library preparations steadily improves metagenomics, there is a growing need to develop fast, reproducible and accurate analytical pipelines for taxonomic classification and quantification. Furthermore, variability in metagenomic library preparation, base calling errors, chimeric reads and nuances in the curation of databases shape considerations for downstream computational analysis that remain challenging [4, 5].

Currently, the two dominant methods for profiling biotic communities are amplicon sequencing and shotgun (i.e. random shearing of whole genomes into small fragments) sequencing. Amplicon sequencing profiles communities by using PCR amplification of target genic sequence(s), typically the rRNA gene. Primers are designed within highly conserved regions that flank regions that are variable between taxonomic groups [6]. Computational analysis of data derived from amplicon sequencing-based libraries is typically limited to genus-level resolution since sequences of high similarity (~97%) are clustered into operational taxonomic units (OTUs) that represent a group of classified and related microbial organisms [7–9]. More recently, the amplicon sequence variant (ASV)-based denoising approaches attempt to obtain species-level profiling [10]. Amplicon sequencing has inherent limitations for drawing functional inferences since accurate quantification is hindered by PCR bias, copy number variation of rRNA genes, oligo design and aggregation abundance estimates at higher taxonomic levels [7, 11–13].

In contrast to amplicon sequencing, shotgun sequencing attempts to holistically evaluate the metagenome and maximize the amount of sequenced genomic material. Tools, such as Kraken2, MetaPhlAn2 and HUManN2 integrate user-directed genome databases that are based on *de novo* assembled metagenomes or curated reference databases [14–16]. The alignment-based approach, adopted by most software, evaluates the similarity of the query to reference sequences using algorithms, such as NCBI BLAST tools. Due to the large datasets generated from next-generation sequencing and the high computational burden associated with MegaBLAST search, tools tend to favor less sensitive and faster alignment-based approaches, e.g. search based on k-mers or use of FM-index [4]. Following alignment search, the best hit approach often suffers from type I errors (false positives) when a query matches multiple best alignment hits derived from several taxa. While the lowest common ancestor (LCA) methods are widely implemented, it addresses this false positive problem by reducing profiling resolution [14, 17, 18]. Similarly, base calling errors can lead to the identification of other closely related taxa that is absent in metagenome but present in a database, hence, the need for stringent read quality filtering.

While shotgun sequencing can improve taxonomic resolution down to strain level, tools often group within-species variants without providing strain taxonomic ID. Since multiple sequence variants can be obtained from a single strain, these discovery-oriented tools (PanPhlAN, StrainPhlAN and DESMAN) would be expected to overestimate strain diversity [2, 19]. For tools that provide strain taxonomic ID (classification-oriented tools: MetaMLST, Pathoscope and StrainEst), aligners used are less sensitive than MegaBLAST and do not require an exact match for strain identification. Tools that map reads to metagenome-assembled genomes (MAGs) reference are limited due to the elimination of genomic variation, which is required to distinguish between strains [5, 20, 21]. Elimination of variants occurs during the collapse of identical contigs, based on the false assumption that mismatch(es) among contigs are sequencing error(s). Even though long-read sequencing can potentially provide enhanced phasing, the current high error rates limit their application for strain-level profiling [22]. On the other hand, tools that map reads to custom minimalist databases or marker gene sets (pan-genes) can be inherently limited [19].

The variability in quality control best practices often necessitates a need for analytical tool benchmarking to determine the reproducibility of results. Besides simulating NGS data of synthetic communities, to more accurately capture the nuance associated with NGS library preparation and sequencing, mock communities can be constituted from microbial cultures. Besides variability in obtaining metagenomic query data, the use of large reference databases (e.g. NCBI databases) creates computational challenges. Furthermore, the presence of genomes of phylogenetically related and environmentally overlapping species/strains in these databases can lead to elevated false positive rates (FPRs). The use of custom minimalist databases decreases computational burden; however, false negative rates (FNRs) are particularly increased in experimental studies where community members are often unknown [23–25].

**Figure 1:**
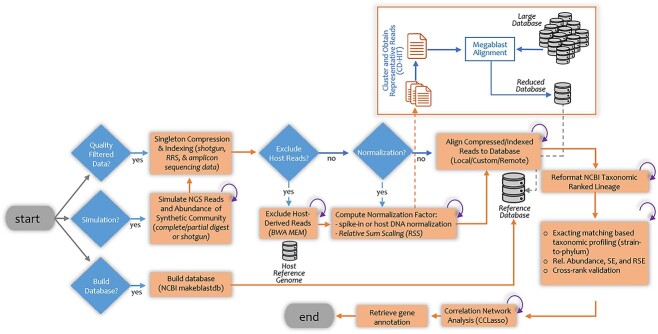
Flow chart of the Qmatey pipeline.

In this study, we present an automated pipeline, Quantitative metagenomic alignment and taxonomic exact matching (Qmatey), that addresses current limitations of metagenomic binning and profiling including poor performance at strain level and when taxa members are genetically similar. To mitigate the slower speed of the more sensitive MegaBLAST alignment tool, we implement an optimized multi-processed MegaBLAST (Figures 1 and S1). We present the utility of Qmatey using multiple databases and three microbiome/metagenome sequencing methods. The metagenomic classification at the strain level and higher taxonomic levels are achieved with an exact matching and exact matching of consensus sequence (EMC) algorithms, respectively. To minimize misclassification and maintain strain level resolution, only taxon-specific diagnostic sequences are used for classification.

## MATERIALS AND METHODS

### Experimental metagenomic communities

Experimental data were obtained from the rhizosphere metagenome of mutant and wild-type maize inbred lines grown under field conditions in 2020 at ETREC, Knoxville, TN. The lesion mimic phenotype caused by an out-of-control hypersensitive response (HR) is driven by an auto-active mutant allele of the Rp1-D disease resistance gene known as Rp1-D21 [26, 27]. The B73-Rp1-D21 and H95-Rp1-D21 mutant lines were created by crossing the Rp1-D21 variants and inbred lines, B73 and H95, respectively. The F1 was subsequently backcrossed to the B73 and H95 parents four times, respectively, while selecting plants that formed spontaneous HR-like lesions. Since Rp1-D21 homozygous plants are not viable, the B73-Rp1-D21 and H95-Rp1-D21 stocks were maintained in a heterozygous state by crossing plants heterozygous for Rp1-D21 as males with their wild-type counterparts as female. Consequently, seeds produced in this way segregate for wild type and mutant phenotype in a ratio of 1:1, respectively. Rhizosphere samples were collected 12 weeks after planting and 3 days after precipitation in the field. Plants within the middle of the plot (to avoid border effect) were carefully dug up with a spade and root segments of about 0.5–1.5 mm in diameter and 10 cm from the tip were collected in a 50 ml falcon tube. To ensure bulk soil is not included in the sample with the rhizosphere, roots were lightly slapped against the spade to remove large and small clumps of soil.

To highlight the compositional data-aware correlation network analyses in a larger population size, a total of 454 sweetpotato F1 individuals and their parents, DM04-001 and Covington, were maintained in a greenhouse (Raleigh, NC). DNA was extracted from young fully developed leaves for the analysis of the leaf-associated metagenome. The parents are contrasting for high and low dry matter, respectively, and for other traits such as flesh color.

### DNA extraction, library preparation and sequencing

Details of the DNA extraction protocol, library preparation and sequencing are provided in the supplementary materials and methods (File S1). The Rp1-D21 maize mutants and wild type were sequenced with 16S amplicon, OmeSeq-quantitative reduced representation (qRRS) and shotgun sequencing methods, while the sweetpotato bi-parental population was sequenced with the GBSpoly method [28].

### MBARC-26 mock community NGS data

The Mock Bacteria ARchaea Community (MBARC-26) is a defined microbial mock community generated through metagenomic NGS released as a resource to benchmark bioinformatic tools [29]. The fastq file for the Illumina HiSeq shotgun sequences of the MBARC-26 mock community was downloaded from the NCBI Sequence Read Archive (SRA ID SRX1836716 and run ID SRR3656745) [29]. Genome assemblies of the 26 species in the MBARC-26 mock community were downloaded from the NCBI genome assembly database to simulate next-generation sequencing within the Qmatey pipeline. Three simulated NGS datasets were generated to mimic mechanical shearing and enzyme-based (complete or partial digestion) fragmentation methods for shotgun and qRRS metagenomic sequencing, respectively.

### NGS data demultiplexing and quality filtering

The sequencing data from all library preparation methods were demultiplexed and quality filtered using ngsComposer, using default parameter settings [30]. These include trimming off the 6 bp buffer sequences, demultiplexing, motif-based read filtering (NsiI/NlaIII and TseI/CviAII for OmeSeq-qRRS and GBSpoly, respectively), quality-based end trimming, adapter contamination removal and finally filtering of reads with >20% of bases with a PHRED quality score that is <20. The MBARC-26 mock community NGS data were quality filtered (i.e. quality-based trimming, adapter removal and read quality filtering) using default parameter settings.

### Overview of Qmatey automated pipeline

Using quality filtered data as input, the Qmatey pipeline completely automates metagenome alignment, taxonomic identification, network correlation analysis and visualization. It is designed to process data derived from various microbiome/metagenome library preparation methods, including amplicon sequencing (16S, ITS and multiplex PCR), shotgun metagenome sequencing and reduced representation sequencing (GBS, RADdseq, ddRADseq and Omeseq-qRRS). Qmatey was run on both experimental (maize rhizosphere and sweetpotato leaves) and synthetic (MBARC-26) NGS data using default parameters. Maize-derived reads were removed by performing alignments with BWA-MEM using the *Zea mays* B73 reference genome version 4. Three sequencing types (OmeSeq-qRRS, WGS and 16S) and four reference databases (nt, RefSeq, 16S and SILVA) were used across our analysis to investigate differences in taxonomic resolution. The MBARC-26 was used for performance testing and demonstration of Qmatey’s ability to both simulate NGS data and perform in-silico enzymatic digestions. Qmatey integrates multiple quality controls to mitigate typical and challenging problems that lead to type I and type II errors in metagenome/microbiome profiling. More detailed information about the Qmatey pipeline is provided in the supplementary methods (File S1).

**Figure 2:**
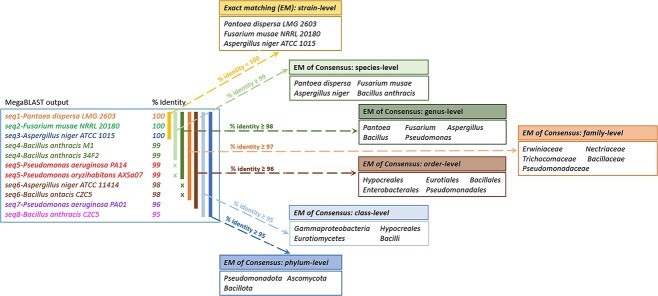
Basis for identifying diagnostic reads at various taxonomic levels within the Qmatey analytical pipeline.

Exact  matching  algorithm  for  identification  of  diagnostic reads: At the core of Qmatey is an exact-matching algorithm for taxonomic binning. This concept relies on ‘diagnostic’ sequences of at least 32 bases within genomes that uniquely identify strains and are also conserved at higher taxa levels. Optionally, a requirement to allow alignments to consume 100% of query length (strain level) and 95–99% of the query length (species-to-phylum level) can be set by specifying the ‘fullqlen_alignment’ parameter as true. The query length of 32 bp required to be consumed during alignment was arbitrarily chosen to account for database issues, such as fragmented assemblies and gaps in assemblies. The exact matching approach prevents retaining of false positive hits, i.e. only 32 bp and full-length diagnostic reads are used. For example, at genus-level profiling, a diagnostic read can match multiple species (*Pseudomonas aeruginosa* and *Pseudomonas putida*) but only within the genus (*Pseudomonas*). If the read matches multiple genera, then it will be discarded (Figure 2). In contrast to other bioinformatic pipelines, each level of taxonomic assignment is done independently and then validated using the *Phylum* results as the ground truth to increase the confidence of lower taxa assignments.

Burn-in MegaBLAST search: To improve speed, depending on the level of diversity in microbiome/metagenome community, a burn-in MegaBLAST search is performed to reduce the size of the database. The burn-in MegaBLAST step (Figure 1) is implemented by clustering reads with the CH-HIT tool [31] at 95% identity threshold and obtaining a representative sequence (subset of total reads), which is then used for the initial burn-in MegaBLAST search at a relaxed percent identity (90%). Subsequently, all the metagenome/microbiome reads are aligned to the identified taxa (reduced database, e.g. small fraction of the NCBI database) during the actual MegaBLAST search. To perform the burn-in MegaBLAST search, the parameter ‘reads_per_megablast_burn_in’ needs to be set to a batch size value (reads per MegaBLAST run) >0 (recommended value = 1000, although the default of 0 indicates no burn-in MegaBLAST search).

### ASV-based microbiome profiling using DADA2

To compare Qmatey’s handling of 16S data, we performed an ASV-based microbiome profiling of the maize rhizosphere 16S sequence data using the R package DADA2 [32]. We followed the standard workflow of filtering for ambiguous bases, primer removal with cutadapt, quality filtering, learning error rates, dereplicating, denoising and merging of paired-end data (minimum overlap of 12 bases and zero mismatches). To assign taxonomy to our ASVs, we used DADA2’s naive Bayesian classifier method, the SILVA nr99 database (version 138.1) and minimum bootstrap confidence of 80% [32, 33]. This approach is comparable to QIIME2 [9].

### K-mer-based metagenomic profiling using kraken2, KrakenUniq and bracken

To compare Qmatey’s performance on the MBARC-26 community, we used the k-mer-based approach implemented in Kraken2 and KrakenUniq for taxonomic classification. To estimate species abundance, Braken was used to compute the proportion of the reads that were mapped to each species [14, 34, 35].

### Performance metrics for benchmarking

Precision was computed by dividing the number of true positive taxa classified correctly by the total number of taxa classified by the tool, i.e. Precision = True Positives/(True Positives + False Positives) [4]. Recall or sensitivity is computed by dividing the number of true positive taxa classified correctly by the total number of taxa in the community, i.e. Recall = True Positives/(True Positives + False Negatives) [4]. We also evaluated the prediction accuracy of the abundance estimates by computing the Pearson correlation coefficients between actual abundance and abundance estimates from the metagenomic profiles.

## RESULTS

### Benchmarking speed performance

To benchmark the speed performance of Qmatey against KrakenUniq [4, 23], the mock community metagenome data (249 465 044 reads) was processed on a compute node with 32 cores and 128 Gb of RAM. Analysis in Qmatey was completed (time taken to run entire pipeline, including compression, and indexing reads) with a runtime of 117 min compared to 93 min for per core KrakenUniq. The MegaBLAST search (burn-in step and actual search) within Qmatey took only 0.0.53 min. Despite using MegaBLAST, which was previously benchmarked to process 5.7 million reads (only 2.3% of the mock data in this study) in 4 h [4], and the full NCBI nt database, Qmatey is only 1.25× slower than KrakenUniq, one of the fastest k-mer-based tools [4].

### Comparison of 16S (DADA2 versus Qmatey), OmeSeq-qRRS and shotgun data

Compared to other tools, Qmatey is robust for all microbiome/metagenome data types and implements both binning and profiling within a single analytical pipeline. Using 16S amplicon data as input, we compared the performance of Qmatey and an ASV-based denoising tool, DADA2. While there are trade-offs between OTU and ASV clustering approaches, they produce similar results. We opted to use the ASV method implemented in DADA2 to produce more precise identification and more details on diversity [32, 33]. Amplicon reads were obtained from the rhizosphere of Rp1-D21 maize mutants and their wild type (B73 and H95).

**Figure 3:**
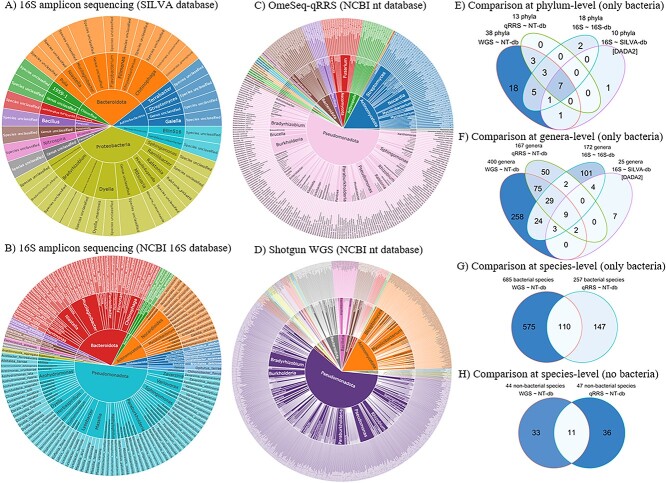
Sunburst showing the diversity of taxa at the species level (from center to outer ring: phylum, genus and species) identified using DADA2 (**A**) and Qmatey (**B)**–(**D**). Venn (**E)**–(**H**) show the overlap of taxa based on a comparison of the three-metagenomic sequencing methods. Sequence data are derived from the rhizosphere of Rp1-D21 mutant and wild-type maize inbred lines.

**Figure 4:**
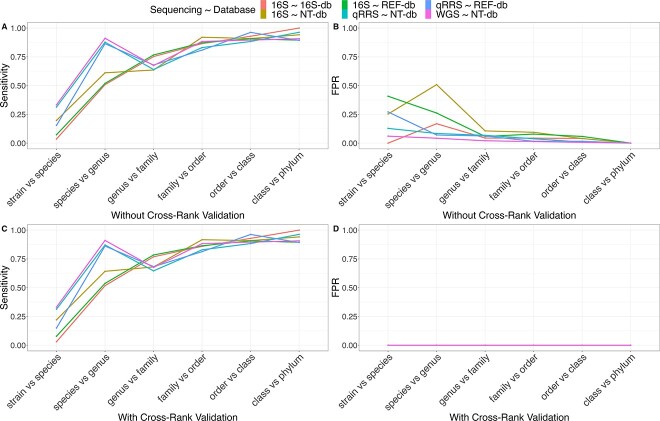
Quality metrics (sensitivity, false positive rate and the number of taxa identified) of metagenomic profiles based on data from the three metagenomic sequencing methods (16S amplicon, OmeSeq-qRRS and shotgun sequencing) and alignments against five databases (NCBI 16S, nt and RefSeq and 16S SILVA database).

Using the NCBI 16S database, Qmatey outperformed DADA2 (using the SILVA 16S database) at all taxonomic ranks and identified 1.8× more phyla, 6.9× more bacterial genera and 43× more bacterial species (Figures 3 and 4). To evaluate congruence, 80% of phyla, 64% of genera and 43% of species identified by DADA2 were validated with Qmatey (Figure 3). While cross-rank validation was used to further reduce FPRs in Qmatey (Figure 4), cross-rank validation could not be performed on the DADA2 result since OTU/ASV-based analyses don’t perform independent analysis at each taxonomic level. Of the three NCBI databases (16S, RefSeq or nt) used for taxonomic assignment in Qmatey, using amplicon data; the nt database identified the highest number of strains and had the highest sensitivity at strain- and species-level, while the RefSeq identified the highest number of species and had the highest sensitivity at the genus level (Figures 4 and S2). The 16S database produced the lowest FPR (Figure 4B).

Analyzing the shotgun and OmeSeq-qRRS metagenome data with Qmatey, 403 and 124 strains were identified, respectively (Figure S3). As expected, the strains found with the 16S data and Qmatey were the only documented strain for their species, hence, effectively species-level resolution. Taxa identified with Qmatey using the shotgun, OmeSeq-qRRS and 16S data include 685 (44 non-bacterial), 257 (47 non-bacterial) and 131 bacterial species, respectively; 400 (38 non-bacterial), 167 (45 non-bacterial) and 172 bacterial genera, respectively; and 38 (15 non-bacterial), 13 (18 non-bacterial) and 18 bacterial phyla, respectively (Figures 2 and S3). In comparison to previous studies of maize rhizospheres, that used the OTU-clustering method, our results are comparable for both composition, diversity and relative abundance [36].

### Quality performance metrics of 16S, OmeSeq-qRRS and shotgun metagenomic data

To assess the performance of Qmatey’s taxonomic profiling algorithm before (Figure 4) and after (Figure S4) cross-rank validation, we computed the FPR and the taxa recovery rate (sensitivity). We computed the FPR based on the percent of taxa found at a lower taxonomic rank but missing in the higher taxonomic rank, while sensitivity is based on the number of taxa found in both lower and higher taxonomic ranks divided by the total number of taxa in the higher rank. The results indicated that independent of the library preparation method, sensitivity increases and FPR decreases at a higher taxonomic rank (Figure 4). OmeSeq-qRRS and shotgun sequencing both had consistently high and similar sensitivity values that outperformed 16S amplicon sequencing at species- and strain-level (up to 10× lower FPR and 25–40% higher sensitivity). Sensitivity at higher taxonomic levels was similar across all sequencing methods (Figure 4A). For sensitivity and FPR, nt outperformed the RefSeq database at the strain level, while there are only marginal differences at other ranks. Sensitivity was the same at the species level for nt and RefSeq database (Figure 4A and B). The same trend was observed for FPR (Figure 4B). For all sensitivity comparisons, cross-rank validation marginally improved sensitivity rates (0–5%) in the rhizosphere experimental dataset (Figure S4).

### Metagenomic profiling of experimental and simulated MBARC-26 mock community

Shotgun data obtained from Illumina NGS short-read sequencing of the MBARC-26 mock community [29] were subjected to three levels of subsampling to capture various proportions of the component genomes, which were consequently used for metagenome profiling (Table 1). Subsampling was based on *in silico* digestion of reads (RE1: NsiI and RE2: NlaIII) and selection of ensuing reads that were flanked by the restriction enzyme motifs (RE1::RE1, RE2::RE2 and RE1::RE2). Metagenome profiling was also performed on reads that were not subsampled. The simulated shotgun NGS reads were subsampled in the same manner (Table 2). To evaluate performance, we use the most important and commonly used metrics for benchmarking (precision and recall).

**Table 1:** Quality metrics of metagenome profiles from Qmatey using MBARC-26 mock community experimental data at various subsampling coverages. Subsampling is based on in silico restriction enzyme (REs: NsiI and NlaIII) fragmentation/trimming followed by the selection of reads flanked by RE motifs and is at least 64 bp long

Taxa level	Stringent quality filtering of reads	Subsampled fragments	Genome coverage (%)	Precision (%)	Recall (%)	Prediction accuracy of abundance	
						Mean read depth^a^	% of total reads^b^
Strain *(Qmatey)*	Yes	*RE1::RE2*	0.24	100	85	0.76	0.74
		*RE1/2::RE1/2*	3.2	100	85	0.68	0.84
		*No subsample*	70.16	96	85	0.91	0.90
	No	*RE1::RE2*	0.87	72	88	0.71	0.81
		*RE1/2::RE1/2*	11.48	9	96	0.68	0.81
Strain *(Kraken2-Braken)*	Yes	*No subsample*	70.16	2	100	–	–
Strain *(KrakenUniq-Braken)*	Yes	*No subsample*	70.16	5	92	–	–
Species *(Qmatey)*	Yes	*RE1::RE2*	0.24	100	92	0.74	0.85
		*RE1/2::RE1/2*	3.2	100	92	0.7	0.88
		*No subsample*	70.16	100	92	0.83	0.93
	No	*RE1::RE2*	0.87	63	96	0.69	0.85
		*RE1/2::RE1/2*	11.48	6	100	0.65	0.84
Species *(Kraken2-Braken)*	Yes	*No subsample*	70.16	1	100	–	0.99
Species *(KrakenUniq-Braken)*	Yes	*No subsample*	70.16	8	100	–	0.99

^a^ *Note:* Abundance estimate is based on average read depth per locus/gene. Required for computing relative abundance across metagenomic profile of multiple samples.

^b^Abundance estimate is based on percentage of the total reads mapped to each taxa. Cannot be used to compute relative abundance across metagenomic profile of multiple samples.

**Table 2:** Quality metrics of metagenome profiles from Qmatey using MBARC-26 mock community simulated NGS data at various subsampling coverages. Subsampling is based on in silico restriction enzyme (REs: NsiI and NlaIII) fragmentation/trimming followed by the selection of reads flanked by RE motifs and is at least 64 bp long

Taxa level	Subsampled	Genome coverage (%)	Fragmentation method (in silico)	Precision (%)	Recall (%)	Prediction accuracy of abundance^a^
Strain *(Qmatey)*	*RE1::RE2*	0.05	Shotgun + complete digest	100	77	0.66
		1.35	Complete digest	100	85	0.38
		97.37	Partial digest	100	96	0.58
	*RE1/2::RE1/2*	1.43	Shotgun + complete digest	100	88	0.5
		20.75	Complete digest	100	92	0.43
		99.52	Partial digest	100	92	0.58
	*No subsample*	84.04	Shotgun	100	81	0.49
Species *(Qmatey)*	*RE1::RE2*	0.05	Shotgun + complete digest	100	92	0.66
		1.35	Complete digest	100	100	0.53
		97.37	Partial digest	100	100	0.58
	*RE1/2::RE1/2*	1.43	Shotgun + complete digest	100	100	0.5
		20.75	Complete digest	100	100	0.42
		99.52	Partial digest	100	100	0.58
	*No subsample*	84.04	Shotgun	100	85	0.52

^a^Note: Abundance estimate is based on average read depth per locus/gene. Required for computing relative abundance across metagenomic profile of multiple samples.

In the experimental and simulated MBARC-26 mock community datasets, overall, the low genome coverage (RE1::RE2) produced better quality metrics than the higher density genome coverages (RE1/2::RE1/2) and no subsampling (Tables 1 and 2). Taxa recovery rate (recall) was better at the species level than at the strain level, while precision was 100% across all the analyses except for no subsampling at the strain level in the experimental data (multiple testing problems associated with using 70% genome coverage) and all cases with lower quality filtering stringency (false positive due to base calling error). The prediction accuracy of abundance in the experimental data was higher than in the simulated data. In the simulation datasets, the prediction accuracy of abundance was highest in the subsampled datasets at low genome coverage at both strain and species levels. On the other hand, the no-subsampled dataset had higher prediction accuracy than the subsampled data.

The rarest community members, *Nocardiopsis dassonvillei* DSM_43111 and *Escherichia coli* K12_MG1655 represent 0.0001% and 0.0019% of the community, respectively, were not recovered under most parameter settings at both strain- and species-level in the experimental data, and at strain level in simulated data (Tables 1 and 2). They were recovered at the species level in the simulated data for most parameter settings (Table 2). They were identified in the MegaBLAST alignment output but did not pass Qmatey’s taxonomic filtering parameters (i.e. no diagnostic read). At strain level in the experimental data, two more rare strains, *Corynebacterium glutamicum* ATCC_13032 and *Salmonella bongori* NCTC_12419 that represent 0.0031% and 0.0015% of the community, respectively, were not recovered. However, bacteriophages annotated to be derived from these species were identified probably because bacteriophages have multiple genome copies per host cell.

### Benchmarking Qmatey against Kraken2-bracken and KrakenUniq-bracken

Benchmarking of Qmatey against the highly ranked (in performance) Kraken2- and KrakenUniq-bracken tools were performed using the shotgun dataset derived from the synthetic mock community. The Bracken tool was used to estimate abundance estimates following Kraken2 and KrakenUniq taxonomic classification. Qmatey performed both classification and profiling (i.e. estimates abundance). Benchmarking against both Kraken2 and KrakenUniq was performed to account for their strengths since Kraken2 is more susceptible to false positives (low precision), hence, limiting interpretation of results. KrakenUniq is supposed to be best suited for reduced false positives and to validate classification [35]. Our benchmarking of Qmatey using metagenomic data with known composition revealed that while Kraken2 and KrakenUniq had slightly higher recall rate at 92–100% (2–4 more taxa than Qmatey), but at a high penalty on precision of 1–8% (i.e. 315–1752 false positive taxa). KrakenUniq only improved precision by 3–7% from the 1–2% precision in Kraken2 (Table 1). On the other hand, Qmatey achieved 100% precision at both species and strain level while maintaining a high recall rate at (i.e. 85% at strain level and 92% at species level).

### Metagenome profiles and correlation network analysis within the context of a holobiont

**Figure 5:**
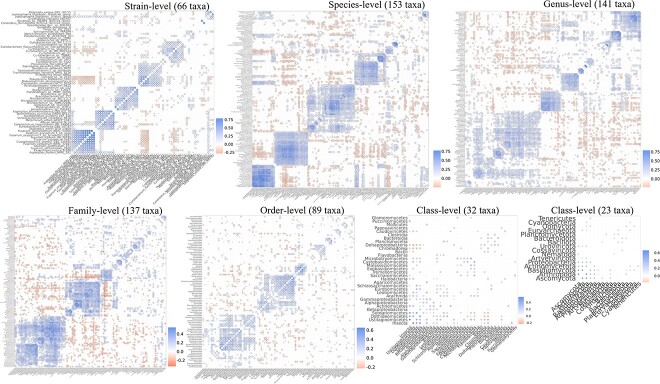
Correlogram plot showing correlation network analysis based on CCLasso-based correlations of leaf-associated metagenomes of a sweetpotato biparental population (DM04-001 × Covington) comprised of 454 F1 progenies and the parents.

The CCLasso-based correlation coefficients computed from metagenomic profiles of the sweetpotato leaf-associated metagenome (two parents and their 454 F1 progenies) revealed biologically relevant taxa (Figures 5 and S5) and microbe-microbe interactions. The CCLasso-based correlation coefficients account for multi-way interactions that underlie each pairwise test. The correlogram plot revealed microbial hubs that comprised strains/species within the same genus (not always the case) or microbes that played overlapping or similar roles within the community, for example, endosymbionts [*Candidatus Hamiltonella defensa (Bemisia tabaci)*, *Rickettsia sp. MEAM1 (B. tabaci)* and *Candidatus_Portiera_aleyrodidarum*] and their insect pest host (*B. tabaci*). Members within microbial hubs were mostly positively correlated, while negative correlations were mostly observed between microbial hubs. In addition, the network analysis revealed that signals defining these microbial hubs degrade from strain to phylum level (Figure 5).

## DISCUSSION

Metagenomics holds great promise for understanding microbe-microbe interactions and the roles they play within physiological, metabolic, ecological and evolutionary (host-microbe-microbe co-evolution) contexts. Nevertheless, routine use of metagenomics suffers from experimental errors, computationally cumbersome analysis of large population-level datasets, low taxonomic resolution, artifacts in databases, high FPRs and the use of curated minimalist databases that don’t represent unknown members of experimental communities [37]. Qmatey addresses these limitations within a completely automated pipeline for ease of use and reproducibility (Table 3). It can use data from various microbiome, metagenome and metatranscriptome sequencing methods as input and provides broad-spectrum taxonomic profiles (viruses-to-eukaryotes). The independent profiling at each taxonomic level allows for the implementation of a new cross-rank validation method that further minimizes FPRs. By partitioning cultured and uncultured strains, Qmatey accounts for the confusion associated with defining the concept of a strain. Its utility for profiling microbes within the context of holobionts and the ability to capture known multipartite interactions underscores its versatility and robustness.

**Table 3:** Strengths and weaknesses of the Qmatey metagenomic classification and profiling pipeline

Strengths
Pipeline is user-friendly, easy to use and produces reproducible results. Performs both taxonomic binning (classification) and profiling (detection and quantification). Analyzes data from various metagenome/microbiome sequencing methods (whole genome shotgun, 16S/ITS amplicon and reduced representation sequencing). Analysis can also exclude highly represented host genome from alignment (increases speed). Strain level classification/profiling with high recall and prediction accuracy of abundance. Identification of diagnostic reads allows for high precision without penalizing recall rate. Flexible for interrogating both large databases and small/custom databases. Also automates building databases from fast sequence with taxa information. Despite the computational intensity of the more sensitive MegaBLAST algorithm, Qmatey is faster than most tools. It improves MegaBLAST speed by hundreds-to-thousands of times. Additionally, subsampling of shotgun data can improve speed by about 1000 times. Multi-node run of a single job submission without the need for a Spark cluster.
Weaknesses *(and approaches in Qmatey to partially mitigate issues).*
Errors, biases, mis-annotation and missing taxa in databases can lead to false positives and false negatives. *Solution: Identification of diagnostic reads can effectively mitigate some database issues, e.g. horizontally transferred genes and contaminating reads in genome assemblies will not be diagnostic and excluded from use in taxonomic classification.* Sequence reads with relaxed quality filtering (i.e. high base calling errors) can lead to false positive rates. *Solution: Increase stringent quality filtering to resolve this issue.*

In this study, we used experimental data obtained from 16S amplicon, OmeSeq-qRRS and shotgun sequencing of the same DNA samples, hence allowing for an unbiased comparison of metagenomic sequencing methods. The datasets revealed that the reference database used for metagenomic alignment and assignment influenced sensitivity and FPR. The more curated RefSeq negatively impacted the sensitivity and FPR, particularly at the strain level. The aim of curating the RefSeq database is to reduce redundancy, but this leads to the exclusion of some taxa and strains, which are more difficult to curate. This explains the lower sensitivity as well as higher FPR (based on cross-rank validation) since curation at a higher taxonomic rank would be more accurate. The less curated nt database produced higher sensitivity, lower FPR and captured viruses that were absent in results obtained with the RefSeq database. Although the curated 16S database had the lowest sensitivity and number of taxa, it had the lowest FPR.

Genome-wide and unbiased sequencing approaches use multiple genes and increase the chance of finding diagnostic reads, particularly in complex metagenomic communities, where very similar strains/species exist within the same community. This underscores the limitation of single gene amplicon sequencing that lacks sensitivity at the strain/species level. The ability to confidently identify taxa at the species level and recover more taxa at the genus level using Qmatey for analyzing 16S amplicon sequencing data highlights the superior approach of exact matching compared to OTU- and ASV-based methods. While ASV-based methods aim to achieve species/strain level identification, denoising and error correction can eliminate true variants, which is evident in the comparison of different error-correction approaches that produce a variable number of ASVs despite using the same dataset [38].

Using the simulated and experimental NGS data from the same MBARC-26 mock community, an unbiased approach can be achieved while benchmarking the performance of Qmatey under ideal and experimental conditions. The latter account for nuances associated with library preparation and sequencing. Comparison of databases provides support for preferentially using the more comprehensive NCBI nt database, particularly at the strain level. This is important since benchmarking efforts often limit the database to known taxa within the community (minimalist databases), hence, leading to biases that overestimate performance metrics. Subsampling of the shotgun data mimics reduced representation sequencing and produced the same recall rate, higher precision at the strain level and comparable prediction accuracies of abundance. The metagenome profiles derived from the experimental and simulated MBARC-26 mock community data were comparable in composition and quality performance metrics. By using the original mock community NGS data and without additional quality filtering, the retained erroneous base calls led to low precision estimates but slightly higher recall that does not justify retaining more reads at the expense of retaining reads/bases with high error rates.

While strain level profiling produces the best resolution and correlation networks that more accurately capture microbial hubs, the requirement to find a diagnostic sequence is limited at strain level since isolate sequencing at some species is limited. Consequently, more species are often found than strains. Species-level profiles might be more appropriate for some downstream analysis; nevertheless, strain identification is crucial for applications, such as validation of interactions, and use as biocontrols, growth-promoting microbes and probiotics. The negative correlations between microbial hubs sometimes revealed known antagonistic interactions between microbes/pathogens and well-documented biocontrols, competitors and plant growth-promoting microbes.

Qmatey’s modularity ensures robust and reproducible metagenomic analysis and provides control over parameters to increase taxonomic precision relative to the accuracy or vice versa. By modifying parameters during the analysis, the user can repeat specific steps in the workflow without repeating time-consuming steps. With a precision and recall of 100% and 85–96%, respectively, Qmatey’s strain-level identification and profiling are remarkable considering most tools produce lower performance metrics at the genus and species level.

**Key Points**

- Qmatey, a completely automated, user-friendly and fast microbiome/metagenome taxonomic binning and profiling software, allows for the reproducible implementation of quality controls and best practices. Given lists of organisms with available reference genomes, it can also automate the simulation of next-generation sequencing of metagenomic libraries.
- Despite interrogating large comprehensive databases and performing read-by-read exact match-based alignments with the computationally intensive and accurate NCBI MegaBLAST tool, Qmatey’s runtime is comparable to some of the fastest tools that use MAGs, curated pan-genes, custom minimalist database and k-mer spectra; approaches that limit taxonomic resolution and sensitivity. In addition, Qmatey can run a job on multiple cluster nodes without using Apache Spark clusters.
- Unlike the LCA approach that reduces taxonomic resolution to address multiple best alignment hits problems, Qmatey maintains strain level resolution and accurate classification/profiling by identifying and using only diagnostic sequences. The exact matching allows the identification of diagnostic reads, resulting in as much as 100% precision (i.e. low FPRs).
- Analyses of experimental data can also be performed within the context of a holobiont, where endophytic and epiphytic microbes constitute a small proportion of the DNA content. Host-derived reads can be masked from analysis to ensure faster analysis.
- Results from Qmatey revealed remarkably high precision (100%) and high recall (85–96%) at the strain level. Most tools produce lower performance metrics than this at the genus and species level.

## Supplementary Material

Fig_S1_bbad351

Fig_S2_bbad351

Fig_S3_bbad351

Fig_S4_bbad351

Fig_S5_bbad351

File_S1_al_Qmatey_Suppl_methods_bbad351

## Data Availability

The Qmatey pipeline is written in bash and R scripting languages (excluding dependencies). It is open source and available on github (https://github.com/bodeolukolu/Qmatey) with a comprehensive set of example datasets obtained from amplicon, shotgun and reduced representation sequencing strategies. All NGS data are publicly available on the NCBI SRA database. These include the 16S, qRRS, and WGS datasets (BioProject ID PRJNA1022580), and the previously published MBARC-26 data [29] (SRX1836716).

## Author Biographies

**Alison Adams** is a postdoctoral research associate in the Department of Plant Pathology, University of Georgia. Her research focuses on biotechnology and omics-based approaches to study aflatoxin issues in peanuts.

**Brandon Kristy** is a Ph.D. student at the Department of Integrative Biology, Michigan State University. His research investigates how below ground microbial interactions can improve the sustainability of perennial bioenergy cropping systems.

**Myranda Gorman** is a veterinary student pursuing a combined DVM-PhD at College of Veterinary Medicine, University of Tennessee. Her interests are in large animal medicine, sustainable agriculture, epidemiology, bacteriology, and bioinformatics.

**Peter Balint-Kurti** is a Research Geneticist with the USDA-ARS located in Raleigh NC on the campus of NC State University. He works on the genetics of disease resistance and defense response in maize.

**Craig Yencho** is a professor at the Department of Horticultural Science, NC State University. He leads global efforts for crop improvement and development of genetics and genomic tools in sweetpotato and potato.

**Bode Olukolu** is an assistant professor at the Department of Entomology and Plant Pathology, University of Tennessee. His research focuses on understanding the genetics of complex traits in maize and polyploid crops.
