# Supplementary figures and images for: Qmatey: an automated pipeline for fast exact matching-based alignment and strain-level taxonomic binning and profiling of metagenomes
Source: Brief Bioinform. 2023 Oct 11;24(6):bbad351. doi: 10.1093/bib/bbad351 (PMC10569747; doi:10.1093/bib/bbad351)

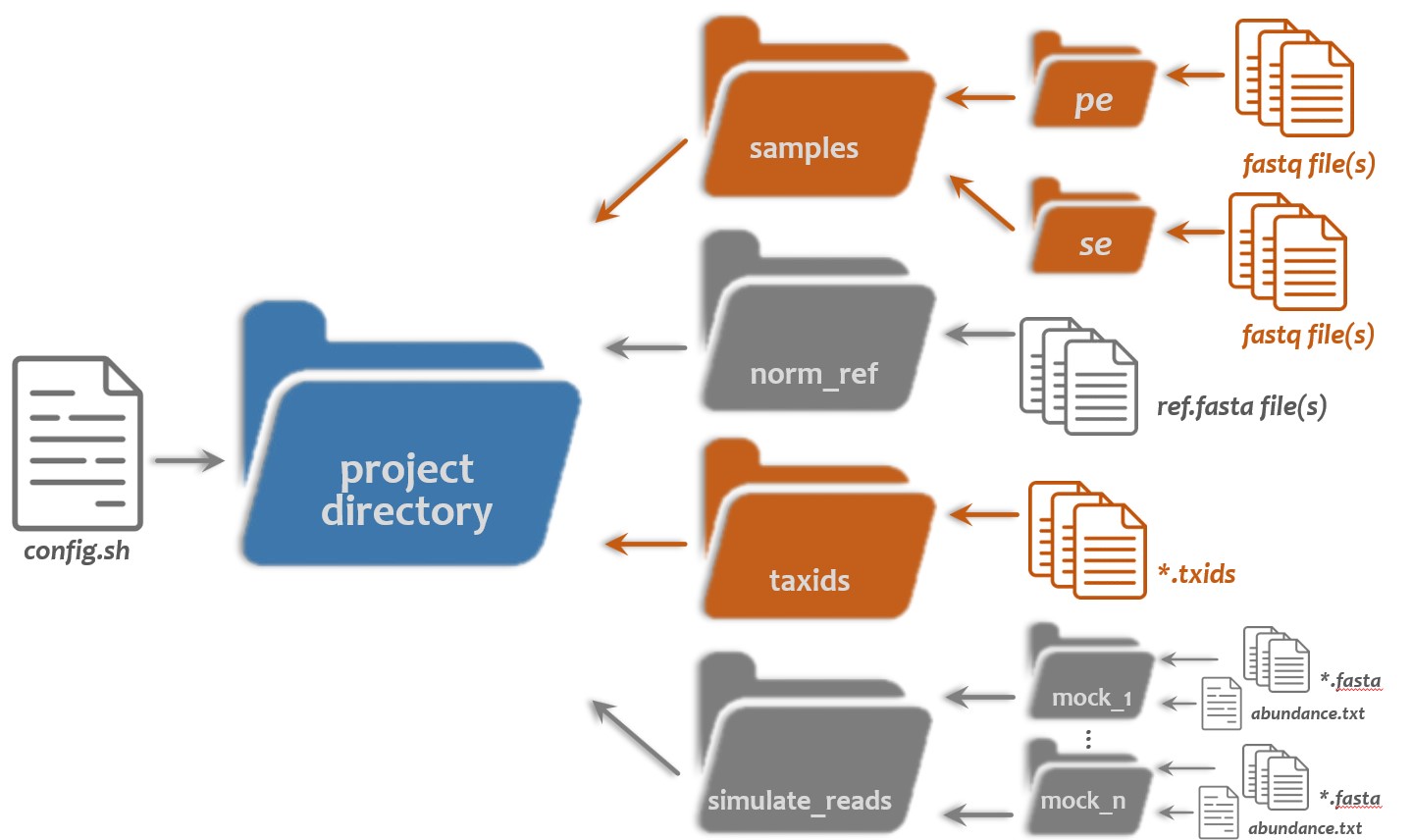

Supplement: Fig_S1_bbad351 [file fig_s1_bbad351.jpeg]

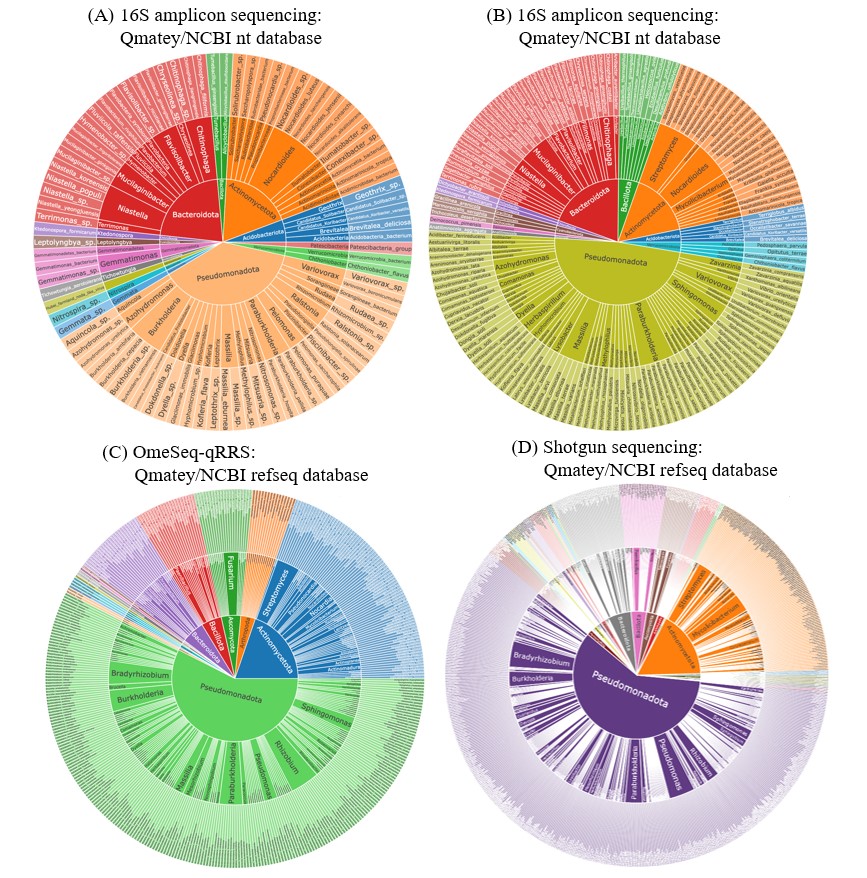

Supplement: Fig_S2_bbad351 [file fig_s2_bbad351.jpeg]

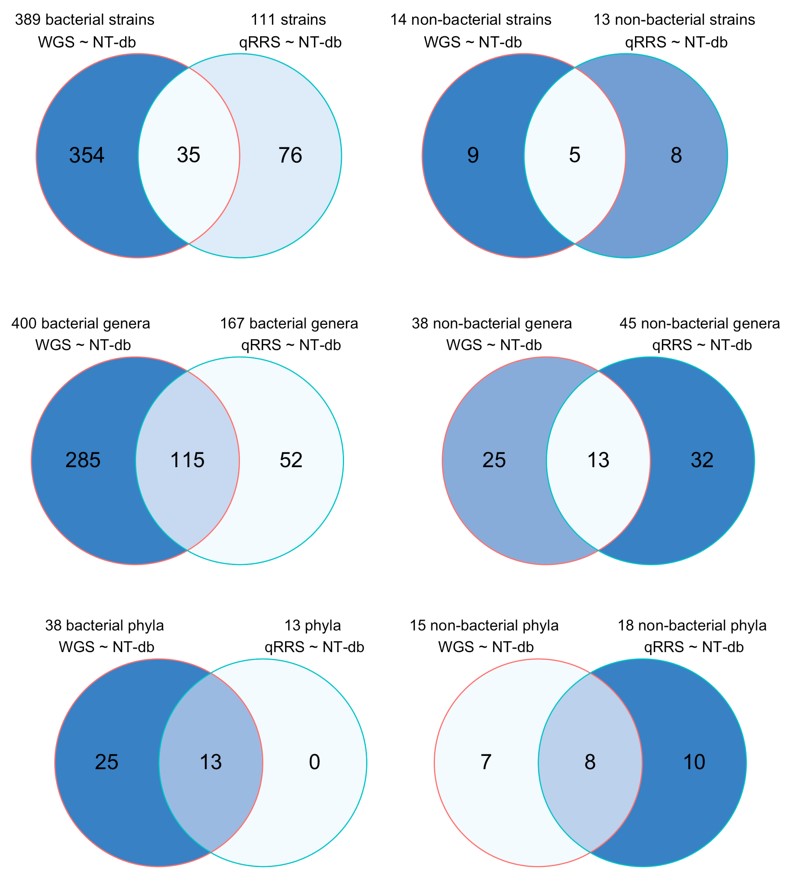

Supplement: Fig_S3_bbad351 [file fig_s3_bbad351.jpeg]

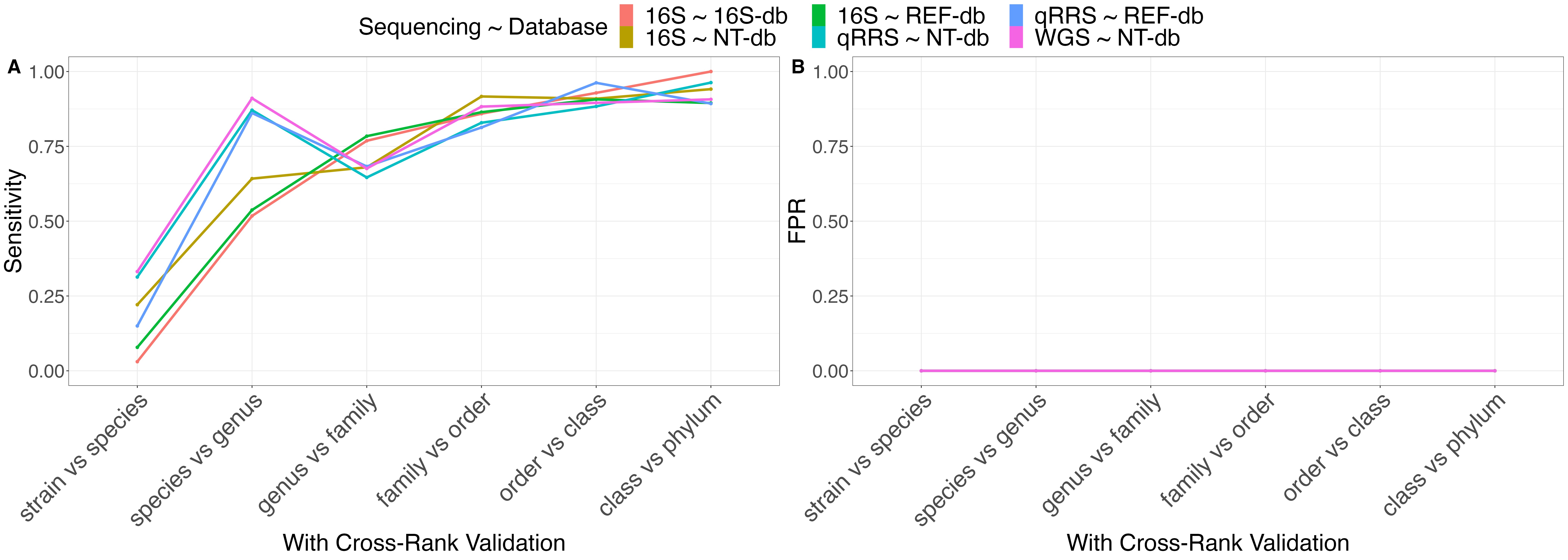

Supplement: Fig_S4_bbad351 [file fig_s4_bbad351.jpeg]

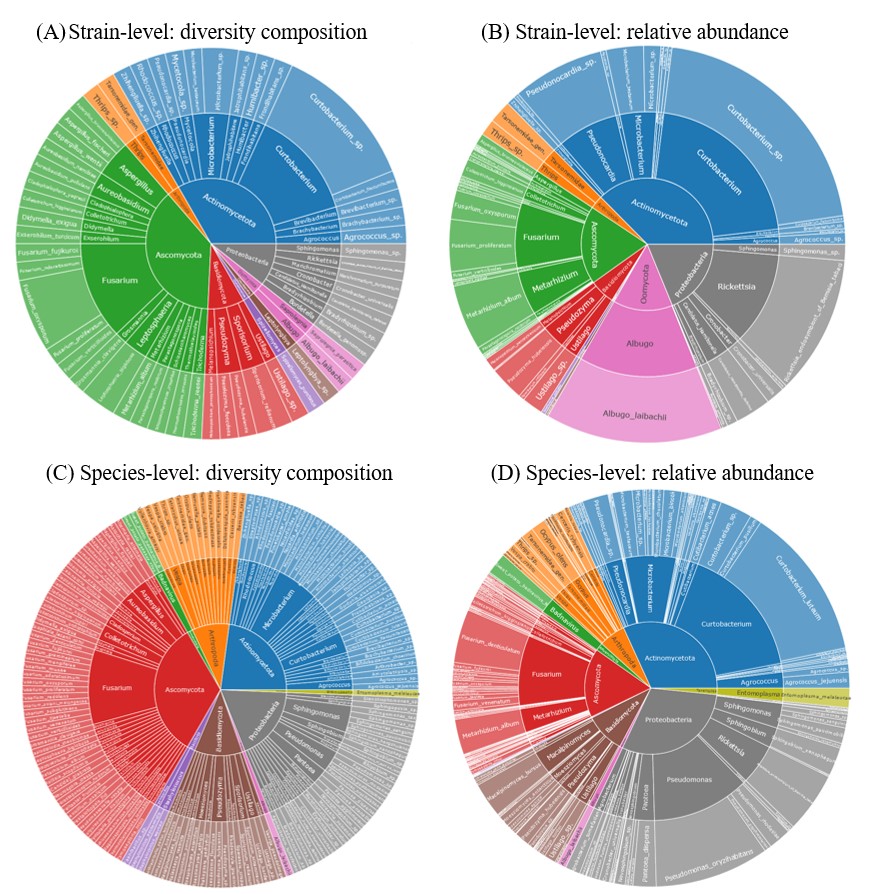

Supplement: Fig_S5_bbad351 [file fig_s5_bbad351.jpeg]
