# Supplementary material for: Qmatey: an automated pipeline for fast exact matching-based alignment and strain-level taxonomic binning and profiling of metagenomes
Source: Brief Bioinform. 2023 Oct 11;24(6):bbad351. doi: 10.1093/bib/bbad351 (PMC10569747; doi:10.1093/bib/bbad351)
Supplement: File_S1_al_Qmatey_Suppl_methods_bbad351 [file file_s1_al_qmatey_suppl_methods_bbad351.docx]

**Supplementary Methods**

*DNA extraction, library preparation, and sequencing.*

DNA extraction from the maize rhizosphere samples was based on a modified SDS-based protocol [1], while DNA from sweetpotato leaves was extracted based on a modified CTAB-based protocol [2]. The DNA samples were quantified with the Invitrogen Quant-iT™ PicoGreen™ dsDNA Assay, normalization to 20 ng/uL, and used for NGS library preparation based on amplicon sequencing (16S sequencing) and genome-wide sequencing (GBSpoly, OmeSeq-qRRS, and shotgun sequencing).

The library preparation of the 16S amplicon sequencing method was performed at the Genomic Sciences Laboratory, NCSU, by targeting the 16S V3 and V4 regions. The PCR primer sequences have been previously published [3]. The Illumina adapter overhang sequences were added to the gene-specific sequences and the full-length forward and reverse primer sequences are TCGTCGGCAGCGTCAGATGTGTATAAGAGACAGCCTACGGGNGGCWGCAG and GTCTCGTGGGCTCGGAGATGTGTATAAGAGACAGGACTACHVGGGTATCTAATCC, respectively. The NGS 16S amplicon sequencing was performed on a MiSeq v3 300x2 PE flow cell. The reduced representation sequencing library preparation (OmeSeq-qRRS patent pending) [4] was performed as previously described [5] and sequenced with the Illumina NovaSeq 6000 150 bp PE S4 flow cell. The GBSpoly library preparation was based on a previous iteration of the OmeSeq-qRRS protocol that used a ligation-based method [6], and the sweetpotato biparental F1 population was sequenced with Illumina HiSeq 2500 125 bp SE. The shotgun sequencing library was prepared with the Illumina Truseq Nano DNA Library Prep Kit and sequenced on a lane of NovaSeq 6000 150 bp PE S4 flow cell.

*Qmatey Pipeline*

Building database: Qmatey uses a single or combination of databases downloaded from the NCBI database (ftp://[ftp.ncbi.nlm.nih.gov/blast/db/](http://ftp.ncbi.nlm.nih.gov/blast/db/)) or other curated databases. Qmatey also builds custom databases as part of the pipeline using the NCBI makeblastdb. To build custom databases with the NCBI makeblastdb, a fasta file containing genome assembly sequences (or unassembled sequences) and a file containing the associated taxa IDs are placed within a user-defined folder that is specified within the config.sh file or upon a prompt during job submission (example files are provided with download).

Simulation of Next-Generation sequencing: Qmatey simulates short- and long-read next-generation sequencing of shotgun and RRS libraries, with genome fragmentation based on in silico random shearing or restriction enzyme digest (complete or partial digest). The expected coverage, E(C), which is the number of bases across genomes covered by at least one sequencing read accounts for discontinuities and position-based sampling biases or edge effect following the extension of the Lander-Waterman equation [7, 8] below:

$$E(C)=2i\left( \lambda-1-\sum_{x=1}^{\lambda-1} e^{-xn/\Pi} \right)+i(\sigma-2(\lambda-1))(1-e^{-n\lambda/\Pi})$$

where *i* is the number of filtered genomic islands, σ is the size of a filtered genomic island, λ is the length of a sequencing read and is less than or equal to σ/2, n is the number of sequencing reads processed, and п is derived from *i*(σ- λ+1) and is the total number of possible placements over whole targets within a library. The term x defines the nucleotide-based island coordinate system, where the origin is the boundary to the left. For a read that no overlapping read will extend beyond the right end of the genomic island, the coordinate range is *x* ∈ {1, 2, 3, ..., *σ* - 2(*λ* - 1)}. For a read in which one or more overlapping reads extend beyond the right end of the genomic island, the coordinate range is *x* ϵ {σ - 2(λ - 1) + 1, …, σ - λ}. For a read that exists on the extreme right of an island, its position is x = σ - λ + 1.

The frequency and span of gaps between genome islands are variable depending on the fragmentation and library preparation method being modeled. Complete and partial digests during RRS can be specified with single or multiple restriction enzymes. Following fragmentation, partially digested and randomly sheared libraries produce multiple partially overlapping and variable length reads that span a genomic island. Reads from completely digested libraries overlap completely and are flushed on both ends for each locus except fragments with indels or erroneous base calls on the distal end of reads. Randomly sheared libraries produce the least number of gaps and provide higher coverage of the genome, while digested libraries produce more gaps since the aim is to sample only a fraction of the genome.

Each genome/taxon in the metagenomic community is replicated to simulate the sequencing coverage for each taxon and consequently the abundance estimate. The mean depth of sequencing coverage is theoretically defined as LN/G, where L is the sequencing read length, N is the total number of reads, and G is the haploid genome size. The abundance of all the taxa in a community is scaled relative to the rarest taxon, hence, the genome of the most abundant taxa might have hundreds to thousands of coverages. Each replicated genome is fragmented independently to simulate the expected random fragmentation. Only fragments within the specified sequence read length range are retained. For restriction enzyme digest-based libraries, only reads flanked by any combination of the restriction site motif(s) on both ends are retained.

Sequence data compression, indexing, and alignment: To improve the speed of MegaBLAST search, joint alignments of reads with 100% full read length match within and across samples were performed following read compression and indexing of read depth and associated sample IDs for each representative read. Since sequence reads are often redundant within and across samples, compression, and indexing of reads are performed before joint alignment, hence speeding up MegaBLAST by several hundreds or thousands of folds depending on the level of read redundancy within and across samples. Subsequently, the read depths of diagnostic sequence reads are used to compute absolute abundance (average of diagnostic sequence read depth), relative abundance estimates (using normalization factor described below), standard error, and relative standard error (RSE). This is different from the ASV-based approach that performs denoising and error correction in other to achieve 100% match across ASV reads. The denoising and error correction can lead to the exclusion of real variants, which is evident in different error correction approaches that produce a variable number of ASVs despite using the same data [9].

Additional speed improvement is achieved by implementing optimized multiprocessing to ensure 80-100% CPU resource allocation to the MegaBLAST tool. Using a multicore processor, a batch of reads is processed on each core. While the number of reads/batches can be user-defined, the number of sequences per batch/core has also been optionally hard-coded for various data types. Genome-wide data alignments are set to a default of 1000 reads/batch. Since 16S/ITS amplicon data are highly conserved and return a lot of hits, only 100 reads/batch. If larger batch sizes are used to increase speed, the RAM can be over-committed and resulting in jobs being killed or chunks of reads completely skipped without outputs. To avoid this, Qmatey is designed to detect this anomaly, kill all processes associated with the job and then provide relevant information in a log file. Consequently, the user can reduce the number of reads per batch and resubmit the job to continue the job while retaining successfully processed batches and removing problematic batches associated with the previous batch size.

The alignments can be performed against the local (recommended) or remote NCBI databases, as well as against custom databases. The MegaBLAST parameters were set to default except for a maximum target sequence of 100,000 (maximum number of aligned sequences to keep), and percent identity of 95%. Some of these thresholds can also be redefined by users with the Qmatey parameters [10]. The high maximum target sequence threshold ensures that all hits are exhaustively sampled from the database. The top best hits can be missed if the threshold is set too low.

Exclusion of host-derived sequencing reads: For endophytic metagenome data derived directly from host tissue, the host reference genome provided in the “norm_ref” folder can be used to exclude reads derived from the host (nuclear, mitochondria, and plastids), which are then excluded from the metagenome analysis. Since the host tissue-associated metagenome can constitute as low as ~2% of total DNA, excluding host DNA will significantly improve analytical speed. Nevertheless, caution should be taken since low-quality host genome assemblies can be contaminated by a significant number of microbial sequences.

Normalization and estimation of relative abundance: To normalize the metagenomic data and compute relative abundance, Qmatey computes normalization factors which are then used to multiply the absolute abundance for each diagnostic read. The normalization can be based on: (i) using the spike-in standard as internal control, preferably recombinant cells that contain unique synthetic tags or organisms that typically do not exist within the metagenomic community [11], (ii) using a host-derived DNA as internal control, where metagenomic DNA is derived from host tissue, and (iii) in the absence of any form of internal control, the less preferred method, the total number of reads from each metagenomic sample can be used. To compute the normalization factor, a novel hybrid approach, Relative Sum Scaling (RSS), is implemented in Qmatey and as an extension of the Trimmed mean of M-values (TMM) and Total Count (TC) methods [12]. The average abundance or relative abundance estimates assumes that each read maps to a unique locus. This is particularly true of genomes composed of mostly single-copy genes/sequences (prokaryotes) and metagenome data, unlike metatranscriptome where abundance varies across the genome based on the deferential gene expression. For metatranscriptome data, the per-read/per-gene abundance estimates would be a more accurate measure rather than the average relative abundance. Like the cumulative sum scaling (CSS) method, the RSS method is robust for metagenomic datasets where high-dimensional sparse data and genome-wide under-sampling (i.e., only diagnostic sequences) are common features [13]. The equation below describes the computation of the normalization factor N*_j_*:

N*_j_* = $S_{j}^{(r)}\sum_{i=1}^{m} Y_{ij}$

where the total read count S is obtained for each sample *j*, a sample r indicating the sample with the lowest total read count that is used as a reference sample, Y*_ij_* is the counts for gene i = 1,..., n, m in sample j = 1, ..., n. To compute normalization factor N*_j_* in experiments with internal controls, like using the host reference, the absolute abundance of the spike-in control is obtained on a sample-by-sample basis using the unique tags of the spike-in controls as the reference sequence. All reads from the spike-in standard DNA can also be used to validate the accuracy of estimating relative abundance by comparing the expected and observed abundance of the taxa within the spike-in standards. On a sample-by-sample basis, all reads are mapped to the reference genomes/sequences (host or spike-in standard) with BWA-MEM and the mapped reads matching host or spike-in control DNA/tags are obtained and used to estimate the normalization factor using the RSS method. The normalization factor is calculated for each sample by dividing the number of reference-aligned reads by the total number of reads in a sample. This ratio is subsequently used as a normalization factor and used to multiply the absolute abundance.

Reformatting the NCBI Ranked Lineage File: The NCBI taxonomic ranked lineage file is required for fetching taxa names and lineage track information from taxid. While this is very useful for metagenomics at various taxonomic rank levels, the curation requires reformatting due to inconsistency in standard nomenclature. The *species* and *genus* column are often designated “NA” when in reality, the *species* and g*enus* name can be extracted from the taxname (“organism name”). Not all entries, particularly viruses and environmental/uncultured organisms, in the ranked lineage follow Linnean binomial nomenclature. Thus, for viruses, species designated “NA” are replaced with the taxname.

Decompressing the combined, compressed, and indexed MegaBLAST results: Upon completion of the MegaBLAST search, entries in the combined and compressed MegaBLAST results are assigned to samples along with the read depth using the index information created during compression. Eventually, two files are generated per sample. Regardless of the library type, the reads and corresponding alignment information are separated into “uncultured” and “cultured”. These files are used to produce two independent metagenomic profiles so that profiles of uncultured microbes can be evaluated separately if it is of interest to users. The theory behind this delineation is to produce metagenomic profiles from only reliable (preferably cultured) sources and well-annotated taxa. The exception to this rule is higher-order eukaryotes and microbes designated *Candidatus* since they are often well-researched and sequenced organisms despite being unculturable for in vitro studies [14]. If a library type is anything other than amplicon, there is a parameter to leave out ribosomal DNA from taxonomic identification and abundance estimation since organisms tend to have a variable number of rDNA sequences in their genomes.

Exact matching-based taxonomic profiling at strain level: Metagenomic reads are stringently filtered for strain-level taxonomic identification. Only reads that match a single taxid at 100% identity and with at least a query coverage of 32 bases or 100% of query length are retained for downstream analysis. Taxonomic rank lineage is obtained from the rank lineage file for each taxonomic ID, while relative abundance and abundance are estimated based on the average read depth of the filtered diagnostic reads associated with each organism. The quantification accuracy for each taxon in each sample is calculated based on the standard error (SE) and relative standard error (RSE) of the read depths of the diagnostic reads. An RSE over 25% is prone to sampling error and should be used with caution.

Taxonomic profiling at species-to-phylum level using the EMC method: The exact matching of consensus sequence algorithm for species- to phylum-level profiling is similar for each level except for the percent identity threshold (i.e., 99% for species, 98% for the genus, 97% for family, 96% for order, and 95% for class and phylum). Like strain level profiling, a minimum query coverage of 32 bases or 95-99 % (species, genus, family, order, class, and phylum at 99, 98, 97, 96, 95 and 95 %, respectively) of query length is also required. Taxonomic rank lineage is obtained from the rank lineage file for each taxonomic ID, and information at lower taxonomic rank is eliminated. Following these, the next step retains only reads that match one or more taxa within a single taxon at the taxonomic rank being profiled. For example, at genus-level profiling, a diagnostic read can match multiple species (*Pseudomonas aeruginosa* and *Pseudomonas putida*) but only within the genus *Pseudomonas*. Abundance, relative abundance, SE, and RSE are computed in a similar manner described above at the strain level.

Controlling for multiple testing problems with cross-rank validation: In a successive manner, starting from strain to class, higher taxonomic ranks are used as a “reference” and the immediate lower rank as the “test”. Taxa in the “test” that is absent in the reference rank are excluded from the results. The results are reported as validated and unvalidated and saved to different directories. Since viruses do not strictly follow the binomial nomenclature, viruses are not cross-rank validated.

Metadata for downstream metatranscriptome analysis: The gene annotation of diagnostic sequences across all samples are retrieved from the MegaBLAST output. This is also provided on a sample-by-sample basis along with the relative abundance of each gene. If the abundance of the genes is derived from metatranscriptome data, this will approximate the gene expression levels rather than the abundance of cells in the community.

Visualizations: Visualization of results include (i) box plots; (ii) sunburst chart showing taxa at user-specified taxonomic ranks; (iii) visualization of the network correlation analysis; and (iv) line plots for sensitivity and false positive rates based on cross-rank validation. The interactive box plots show the distribution of the number of unique diagnostics reads, abundance, relative abundance, standard error, and relative standard errors for each taxon (color-coded based on phylum) across samples. The plotted values are based on averages computed across diagnostic reads per taxa and per sample. Generated using the R package plotly [15], a minimum of 3 samples are required for these boxplot visualizations. Two versions of the interactive sunburst chart, using the R package plotme ​​(https://github.com/yogevherz/plotme), are metagenomic profiles at each taxonomic rank to visualize the diversity (unweighted) and abundance (weighted by relative or absolute abundance estimate). The sunburst default shows 2 or 3 layers/circles, which include the outer (only at strain- and species-level; default is species), middle (default is genus), and inner (default is phylum) circle. The correlogram is based on the correlation coefficients computed using the CCLasso method [16], which accounts for the multi-way interactions in the compositional data. A minimum of 24 samples are required for the correlation analysis and visualization. Multiple correlograms are produced based on combinations of positive and negative correlation coefficients. Multiple versions of the correlograms are also created based on as well as based on the thresholds for limiting the level of zero-inflation (e.g., a 20% threshold, only taxa with relative/absolute abundance greater than 0 in a minimum of 20% of samples are retained for analysis). Multiple versions of the boxplots and sunbursts are also created with different levels of zero-inflation. Sensitivity and false positive rates are computed to show trends across taxonomic ranks before and after cross-validation.

**References**

1. Pang MA, N.; Lee, C. W.; and Ng C. C. Isolation of High Molecular Weight DNA from Forest Topsoil for Metagenomic Analysis. Asia Pacific Journal of Molecular Biology and Biotechnology 2008;16(2):35-41.

2. Doyle JJ, Doyle JL. A rapid DNA isolation procedure for small quantities of fresh leaf tissue. Phytochem Bull. 1987;19:11-5.

3. Klindworth A, Pruesse E, Schweer T, et al. Evaluation of general 16S ribosomal RNA gene PCR primers for classical and next-generation sequencing-based diversity studies. Nucleic Acids Res. 2013;41(1).

4. Olukolu BA, Yencho GC, inventors; North Carolina State University and University of Tennessee, Knoxville, assignee. Compositions and methods related to quantitative reduced representation sequencing. USA2020.

5. Kuster RD, Yencho GC, Olukolu BA. ngsComposer: an automated pipeline for empirically based NGS data quality filtering. Briefings in Bioinformatics. 2021;22(5).

6. Wadl PA, Olukolu BA, Branham SE, et al. Genetic Diversity and Population Structure of the USDA Sweetpotato (Ipomoea batatas) Germplasm Collections Using GBSpoly. Front Plant Sci. 2018;9:1166.

7. Lander ES, Waterman MS. Genomic mapping by fingerprinting random clones: a mathematical analysis. Genomics. 1988;2(3):231-9.

8. Wendl MC, Barbazuk WB. Extension of Lander-Waterman theory for sequencing filtered DNA libraries. Bmc Bioinformatics. 2005;6.

9. Nearing JT, Douglas GM, Comeau AM, et al. Denoising the Denoisers: an independent evaluation of microbiome sequence error-correction approaches. Peerj. 2018;6.

10. Morgulis A, Coulouris G, Raytselis Y, et al. Database indexing for production MegaBLAST searches. Bioinformatics. 2008;24(16):1757-64.

11. Hardwick SA, Deveson IW, Mercer TR. Reference standards for next-generation sequencing. Nat Rev Genet. 2017;18(8):473-84.

12. Pereira MB, Wallroth M, Jonsson V, et al. Comparison of normalization methods for the analysis of metagenomic gene abundance data. Bmc Genomics. 2018;19.

13. Paulson JN, Stine OC, Bravo HC, et al. Differential abundance analysis for microbial marker-gene surveys. Nat Methods. 2013;10(12):1200-+.

14. Pallen MJ. The status Candidatus for uncultured taxa of Bacteria and Archaea: SWOT analysis. Int J Syst Evol Micr. 2021;71(9).

15. Li R, Bilal U. Interactive web-based data visualization with R, plotly, and shiny. Biometrics. 2021;77(2):776-7.

16. Fang HY, Huang CC, Zhao HY, et al. CCLasso: correlation inference for compositional data through Lasso. Bioinformatics. 2015;31(19):3172-80.
